# DNA-demethylating and anti-tumor activity of synthetic miR-29b mimics in multiple myeloma

**DOI:** 10.18632/oncotarget.675

**Published:** 2012-10-21

**Authors:** Nicola Amodio, Marzia Leotta, Dina Bellizzi, Maria Teresa Di Martino, Patrizia D'Aquila, Marta Lionetti, Fernanda Fabiani, Emanuela Leone, Anna Maria Gullà, Giuseppe Passarino, Michele Caraglia, Massimo Negrini, Antonino Neri, Antonio Giordano, Pierosandro Tagliaferri, Pierfrancesco Tassone

**Affiliations:** ^1^ Department of Experimental and Clinical Medicine, Magna Graecia University and Medical Oncology Unit, T. Campanella Cancer Center, Salvatore Venuta University Campus, Catanzaro, Italy; ^2^ Department of Cell Biology, University of Calabria, Cosenza; ^3^ Department of Medical Sciences University of Milan, Hematology 1, IRCCS Policlinico Foundation, Milan, Italy; ^4^ Department of Biochemistry and Biophysics, Second University of Naples, Naples, Italy; ^5^ Department of Experimental Medicine and Diagnostics, University of Ferrara; ^6^ Sbarro Institute for Cancer Research and Molecular Medicine, Center for Biotechnology, College of Science and Technology, Temple University, Philadelphia, PA, USA

**Keywords:** miR-29b, microRNA, multiple myeloma, DNA methyltransferases, DNMT

## Abstract

Aberrant DNA methylation plays a relevant role in multiple myeloma (MM) pathogenesis. MicroRNAs (miRNAs) are a class of small non-coding RNAs that recently emerged as master regulator of gene expression by targeting protein-coding mRNAs. However, miRNAs involvement in the regulation of the epigenetic machinery and their potential use as therapeutics in MM remain to be investigated. Here, we provide evidence that the expression of de novo DNA methyltransferases (DNMTs) is deregulated in MM cells. Moreover, we show that miR-29b targets DNMT3A and DNMT3B mRNAs and reduces global DNA methylation in MM cells. In vitro transfection of MM cells with synthetic miR-29b mimics significantly impairs cell cycle progression and also potentiates the growth-inhibitory effects induced by the demethylating agent 5-azacitidine. Most importantly, in vivo intratumor or systemic delivery of synthetic miR-29b mimics, in two clinically relevant murine models of human MM, including the SCID-synth-hu system, induces significant anti-tumor effects. All together, our findings demonstrate that aberrant DNMTs expression is efficiently modulated by tumor suppressive synthetic miR-29b mimics, indicating that methyloma modulation is a novel matter of investigation in miRNA-based therapy of MM.

## INTRODUCTION

Multiple myeloma (MM) is a clonal malignancy of bone marrow plasma cells that develops as a consequence of a multistep transformation process [1-3]. Several genetic lesions associated to the activation of signal transduction pathways, which stimulate MM cell survival and proliferation, have been elucidated in the past decade [1-7]. Presently, there is growing evidence that epigenetic events play a major role in MM pathogenesis [8, 9]. Changes in DNA methylation are key epigenetic features known to regulate gene expression. In mammalians, DNA methylation is characterized by an enzymatic addition of a methyl group at the carbon 5 position of cytosine in the context of the sequence 5' cytosine-guanosine (CpG) through DNA methyltransferase (DNMTs) activity[9]. CpG dinucleotides are clustered in regions, named CpG-islands, present in almost 60% of all gene promoters [10]. Once hyper-methylated, CpG-islands mediate gene-transcriptional silencing [11]. DNMT isoforms, including DNMT1, DNMT3A and DNMT3B, have distinct roles in genomic methylation[12]: DNMT3A and DNMT3B are responsible for *de novo* DNA methylation, whereas DNMT1 accounts for replicating the DNA methylation pattern in genomic DNA [13]. A number of studies suggest that DNMT genes are frequently overexpressed in human cancer and in the cell transformation process [14-17], though mutations of DNMT genes might also occur [18]. DNMTs overexpression is produced by different mechanisms, including aberrant cell cycle control, increased mRNA and protein stability, and E2-F-mediated DNMTs promoter activation [19, 20]. Most importantly, silencing of tumor suppressor genes by aberrant DNA hyper-methylation has been reported in hematologic malignancies, including acute myeloid leukemia [21] and multiple myeloma [8, 22].

Recent evidence supports a role for microRNAs (miRNAs) as relevant players in aberrant mechanisms of DNA hyper-methylation [23, 24]. MiRNAs are an evolutionarily conserved class of small non-coding RNAs (20-24 nucleotides) that regulate gene expression via complete or partial matching to target genes at the 3' untranslated region (UTR), causing suppression of protein translation or messenger RNA (mRNA) degradation [25]. To date, no evidence of miRNAs involvement in antagonizing aberrant methylation in MM has been reported. Moreover, although their involvement in the pathogenesis of MM has been underlined by several observations, only few miRNAs have been evaluated as therapeutic agents in the treatment of this disease [26, 27].

In the present study, we evaluated whether miR-29b could inhibit *de novo* DNMTs expression. Moreover, synthetic miR-29b oligonucleotides formulated with a novel Neutral Lipid Emulsion (NLE) [28] delivery system were used to evaluate the effect of miR-29b in different and clinically relevant murine xenograft models of human MM, including the most innovative SCID-*synth-hu* system [29], which recapitulates the growth of human MM cells within the human bone marrow microenvironment (huBMM)[30].

## RESULTS

### Expression of DNMT3A and DNMT3B in MM primary samples and cell lines

**Figure 1:**
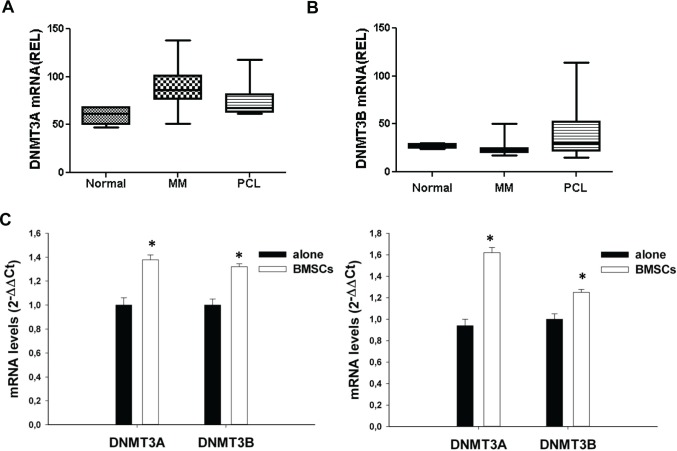
Expression of DNMT3A and DNMT3B in MM and PCL patients or in MM cell lines Differential expression of DNMT3A (A) and DNMT3B (B) in controls, MM and PCL patients. DNMT3A and DNMT3B mRNA levels were obtained by cDNA microarray and reported as raw expression values. The statistical significance of differences among the groups was assessed using Kruskal-Wallis test (P=0,0018 for DNMT3A; P=0,05 for DNMT3B). (C). Quantitative RT-PCR of DNMT3A or DNMT3B in KMS12 (left panel) and NCI-H929 (right panel) cells co-cultured with MM patient-derived BMSCs and then immunopurified by immunomagnetic sorting with anti-CD138 beads. Raw Ct values were normalized to GAPDH and expressed as ΔΔCt values calculated using the comparative cross threshold method. DNMT3A or DNMT3B levels in cells cultured in absence of BMSCs were set as internal reference. Data are the average of two independent co-culture experiments performed in triplicate. P values were obtained using two-tailed *t* test. * P<0,01.

We first evaluated the expression of DNMT3A, DNMT3B and DNMT1 mRNAs on a dataset of high-density cDNA-microarrays of primary CD138^+^ cells from intramedullary MM (n=55) or PCL (n=5) patients and from normal healthy donors (PCs, n=4). As compared to normal CD138^+^ cells, PCs from MM and PCL patients showed higher expression of DNMT3A (Fig. 1A), and, at a lesser extent, of DNMT3B mRNAs (Fig. 1B), suggesting a potential role of *de novo* DNMTs in malignant transformation; conversely, DNMT1 levels were lower in cancer cells compared to normal PCs (Supplemental Fig. 1). To determine whether the bone marrow microenvironment (BMM), which triggers the proliferation and supports the survival of MM cells, could affect *de novo* DNMTs expression, KMS12 and NCI-H929 MM cell lines were cultured for 12 or 24 hours adherent to bone marrow stromal cells (BMSCs) and DNMT3A and DNMT3B levels were then analyzed by q-RT-PCR (Fig. 1C). Interestingly, adhesion of MM cells to BMSCs induced up-regulation of DNMT3A and DNMT3B mRNA levels in both MM cell lines, suggesting that the BM *milieu* might influence DNA methylation of MM cells.

### miR-29b targets de novo DNMTs in MM cells

**Figure 2:**
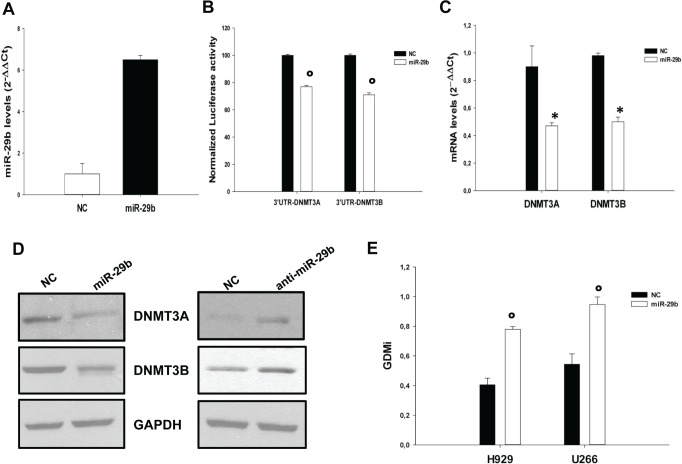
MiR-29b targets DNMT3A and DNMT3B and reduces global DNA methylation levels in MM cells (A). Quantitative RT-PCR of miR-29b levels in INA-6 cells transfected with synthetic miR-29b mimics or scrambled oligonucleotides (NC). Raw Ct values were normalized to RNU44 housekeeping snoRNA and expressed as ΔΔCt values. MiR-29b levels in cells transfected with NC were set as internal reference. Data are the average of two independent transfection experiments performed in triplicate.(B). Dual luciferase assay of INA-6 cells co-transfected with firefly luciferase constructs containing the 3'UTR of DNMT3A or DNMT3B and miR-29b or scrambled oligonucleotides (NC) as indicated. The firefly luciferase activity was normalized to renilla luciferase activity. The data are shown as relative luciferase activity of miR-29b-transfected cells as compared to the control (NC) of a total of six experiments from three independent transfections. °P<0,01. (C). Quantitative RT-PCR of DNMT3A and DNMT3B 24 hours after transfection with synthetic miR-29b or scrambled oligonucleotides (NC) in INA-6 cells. The results are shown as average mRNA expression, in three independent experiments, after normalization with GAPDH and ΔΔCt calculations *P<0,05. (D). Immunoblot of DNMT3A and DNMT3B 24 hours after transfection of INA-6 with synthetic miR-29b or scrambled oligonucleotides (left panel) or in SKMM1 cells transduced with antagomiR-29b (anti-miR-29b) or the empty vector (right panel). GAPDH was used as loading control. (E). GDMi values of U266 and NCI-H929 cells transfected with synthetic miR-29b mimics or scrambled oligonucleotides (NC). The values represent the main of three independent triplicate experiments with standard error mean. °P<0,01.

*In silico* search for target prediction [31, 32] indicates that both DNMT3A and DNMT3B are *bona fide* targets of miR-29b. To validate this interaction in MM cells, INA-6 cells were co-transfected with synthetic miR-29b or scrambled oligonucleotides (NC), together with an expression vector carrying the 3'UTR of DNMT3A or DNMT3B mRNA cloned downstream to the luciferase reporter gene. Figure 2A shows miR-29b levels after transfection. A significantly lower luciferase activity in INA-6 cells transfected with miR-29b mimics as compared to control was detected (Fig. 2B). Consistently, miR-29b transfection reduced DNMT3A and DNMT3B mRNA (Fig. 2C) and protein levels (Fig. 2D, left panel), as assessed by q-RT-PCR and western blotting analysis. On the basis of these findings we investigated whether miR-29b inhibition could up-regulate *de novo* DNMTs expression. SKMM1 cells were stably transduced with a lentiviral vector carrying the antagomiR-29b; miR-29b levels after transduction are reported in Supplemental Fig. 2. Of note, miR-29b inhibition indeed led to up-regulation of both DNMT3A and DNMT3B protein levels, as assessed by western blotting analysis (Fig. 2D, right panel). It was possible to conclude that miR-29b is a direct regulator of DNMT3A and DNMT3B expression in MM cells. These findings also suggested that miR-29b could induce epigenetic modifications in cancer cells. To address this issue, we transfected either NCI-H929 or U266 MM cells with miR-29b synthetic mimics or scrambled oligonucleotides and the global methylation levels were measured by a well established luminometric method [33]. MiR-29b levels after transfection are reported in supplemental Fig. 3. As shown in Fig. 2E, miR-29b transfection resulted in a robust reduction of the global methylation levels of MM cell lines, thus supporting its role in the epigenetic control of MM cells.

### Inverse correlation between miR-29b and DNMT3B in MM cell lines

**Figure 3:**
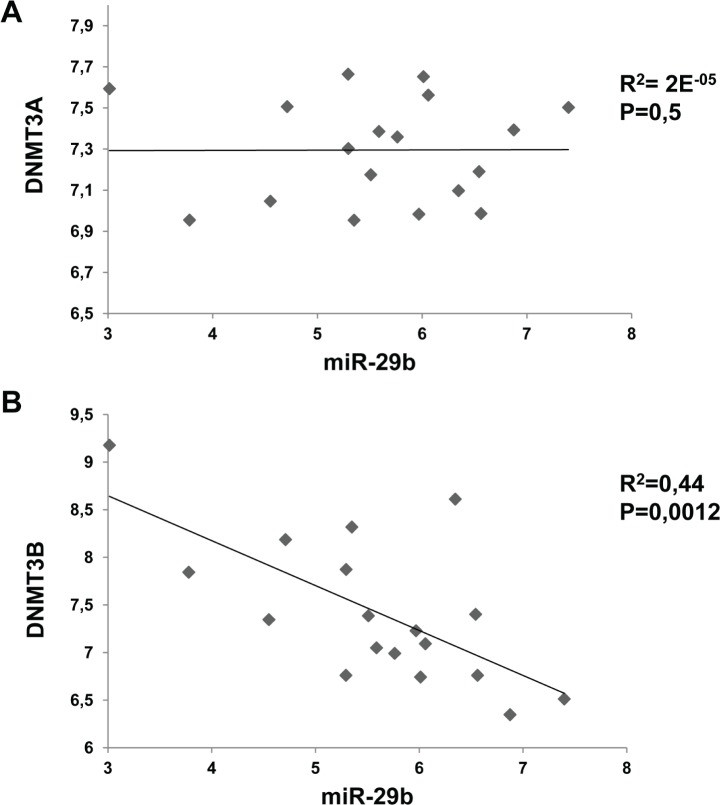
Inverse correlation between miR-29b and DNMT3B levels in MM cell lines Correlation of endogenous miR-29b levels with DNMT3A (A) and DNMT3B (B) mRNA levels determined by high density microarray of mRNA and miRNA expression in a panel of 17 MM cell lines. Log values of raw data are reported in graph. R= regression coefficient.

To assess the relevance of the interaction between miR-29b and *de novo* DNMTs, we analyzed the correlation between miR-29b and DNMT3A or DNMT3B mRNA levels in a panel of 17 MM cell lines. Raw microarray expression levels for DNMT3A, DNMT3B and miR-29b are reported in Supplemental Fig. 4. Notably, such integrated analysis highlighted an inverse correlation between miR-29b and DNMT3B expression levels (p=0,0012), whereas no correlation between miR-29b and DNMT3A could be demonstrated (Fig. 3).

### Synthetic miR-29b mimics impair cell cycle progression and potentiate 5-azacitidine effects

**Figure 4:**
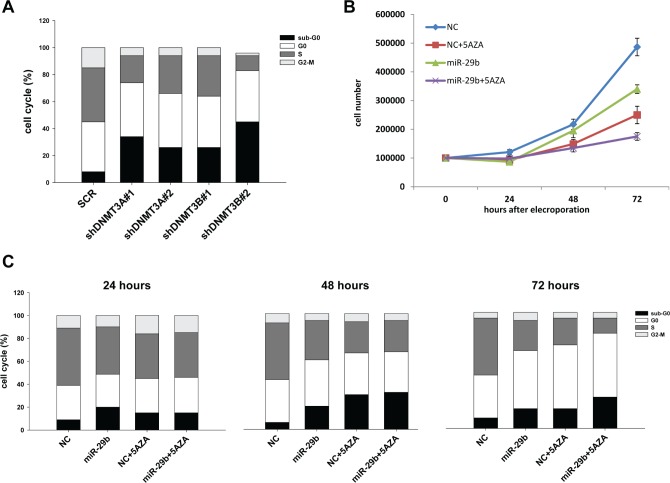
MiR-29b alters cell cycle progression and potentiates 5-azacitidine effects in MM cells (A). Cell cycle analysis of NCI-H929 cells transduced with two different shRNAs against DNMT3A or DNMT3B or with a vector carrying a scrambled sequence (SCR). At least 20000 events for each point were analyzed in three independent experiments. Results are representative of one out of three experiments. (B). Cell growth curves of NCI-H929 cells transfected with synthetic miR-29b (miR-29b) or scrambled oligonucleotides (NC) with 5μM azacitidine (5-AZA) or vehicle (RPMI medium). Averaged values of three independent experiments are plotted including ±SD. P values 72hours after electroporation were obtained using two-tailed *t* test (P= 0,0039 for NC vs miR-29b; P=0,0028 for NC+AZA vs miR-29b+AZA). (C). Cell cycle analysis of NCI-H929 cells transfected with synthetic miR-29b mimics or scrambled oligonucleotides (NC) and then treated with 5μM azacitidine (5-AZA) or vehicle for 24, 48 or 72 hours. Results are representative of one out of three independent experiments.

We subsequently studied *in vitro* effects produced by targeting *de novo* DNMTs in MM cells. In details, NCI-H929 MM cells were stably transduced with two different shRNAs against DNMT3A or DNMT3B or with a scrambled control. After lentiviral transduction, cells were selected with puromycin and levels of DNMT3A and DNMT3B mRNAs were analyzed by q-RT-PCR (Supplemental Fig. 5). Interestingly, *de novo* DNMTs silencing significantly altered cell cycle of MM cells, as revealed by both the strong decrease in S-phase and the relevant increase in hypodiploid cells, indicative of apoptosis, in cells silenced for DNMT3A and DNMT3B compared to scrambled-transduced cells (Fig. 4A). These findings indicate that *de novo* DNMTs represent promising targets for therapeutic intervention in MM. Then, we studied the effects induced by synthetic miR-29b mimics, alone or in combination with the demethylating agent 5-azacitidine, on cell growth and cell cycle regulation of MM cells. *In vitro* transfection of synthetic miR-29b mimics decreased cell growth in a time-dependent manner and potentiated 5-azacitidine anti-proliferative effects at 72 hours (percentage of growth inhibition at 72 hours were 45%, 35% and 65% for miR-29b, 5-azacitidine and combination treatment respectively; Fig. 4B). Cell cycle analysis revealed that miR-29b induced apoptosis, as assessed by the increase of hypodiploid cell percentage, to an extent similar to that caused by 5-azacitidine; the combined treatment produced, at 72 hours, an increase of apoptosis and decrease in S-phase as compared to miR-29b or 5-azacitidine treatment alone (Fig. 4C). These results suggest that the potentiation on cell growth inhibition is likely due to the perturbation of cell cycle distribution and to the increase of apoptosis occurrence.

### Synthetic miR-29b mimics exert anti-MM activity in vivo

**Figure 5:**
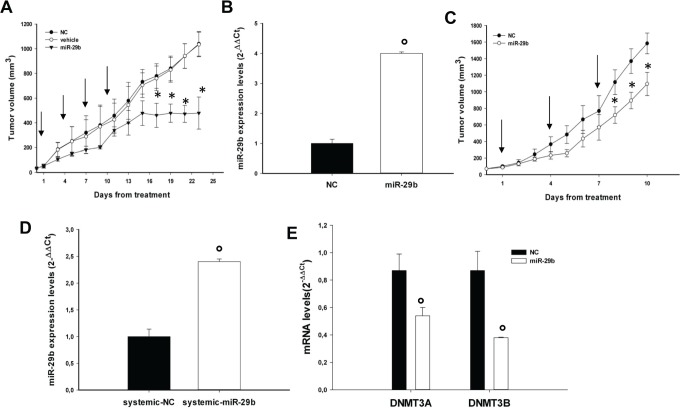
In vivo anti-tumor activity of miR-29b mimics after intratumoral or systemic delivery in MM mouse-models (A). *In vivo* tumor growth of SKMM1 xenografts intratumorally-treated with NLE (MaxSuppressor^TM^ In Vivo RNA-LANCER II)-miR-29b or controls. Palpable subcutaneous tumor xenografts were treated every 3 days (indicated by arrows) for a total of 4 injections, with 20 μg of formulated miR-29b or miR-NC (NC). As control 2 separate groups of tumor–bearing animals were injected with vehicle alone or NLE-formulated scrambled oligonucleotides (NC). Tumors were measured with an electronic caliper every 3 days, averaged tumor volume of each group ±SD are shown. P values were calculated for miR-29b *versus* miR-NC. *P<0.001. (B). Quantitative RT-PCR of miR-29b levels in retrieved xenografts after intratumor injection of miR-29b mimics or scrambled oligonucleotides. Raw Ct values were normalized to RNU44 housekeeping snoRNA and expressed as ΔΔCt values. Data are the average of two independent triplicate experiments performed on two NC and two miR-29b injected animals °P<0,01. (C). *In vivo* tumor growth of OPM1 xenografts after systemic delivery of miR-29b or scrambled oligonucleotides (NC). Mice carrying palpable subcutaneous OPM1 tumor xenografts were treated with 20 μg of NLE-formulated miR-29b or scrambled oligonucleotides (NC) by intravenous tail vein injections (arrows indicate the day of injection). Caliper measurement of tumors were taken every day from the day of first treatment. Averaged tumor volumes of mice are reported ± SE. *P<0.05). (D). Quantitative RT-PCR of miR-29b levels in retrieved xenografts after system injection of miR-29b mimics or scrambled oligonucleotides (NC). Data are the average of two triplicate experiments performed on two NC and two miR-29b injected animals. °P<0,01. (E). Quantitative RT-PCR of DNMT3A or DNMT3B in retrieved xenografts after system injection of miR-29b mimics or scrambled oligonucleotides (NC). Raw Ct values were normalized to GAPDH and expressed as ΔΔCt values calculated using the comparative cross threshold method. DNMT3A or DNMT3B levels in NC-tumors were set as internal reference. Data are the average of two independent triplicate experiments performed on two NC and two miR-29b injected animals. °P<0,01.

The effect of miR-29b on MM cell growth *in vivo* was evaluated in 2 different experimental systems. In a first model, we explored the *in vivo* anti-tumor potential of miR-29b on MM xenografts in SCID mice by intratumor delivery of synthetic miR-29b-mimics. SKMM1 MM cells were injected in a cohort of 15 mice and when tumors became palpable, mice were randomized into 3 groups and treated intratumorally with synthetic miR-29b mimics, miR-NC or vehicle alone. To achieve an efficient delivery of miR-29b or miR-NC, we formulated synthetic miR-29b-mimics with NLE particles [28], a novel *in vivo* delivery system for oligonucleotides. As shown in Fig. 5A, repeated intratumor injection of NLE-formulated synthetic miR-29b (1mg/Kg; 5 injections, 3 days apart), significantly inhibited growth of MM xenografts. Our findings demonstrate that the delivery of synthetic miR-29b mimics exerts anti-MM activity *in vivo*. Importantly, the growth-inhibitory effect well correlated with miR-29b accumulation in tumor tissues (Fig. 5B), as assessed by q-RT-PCR in retrieved xenografts. We next explored the potential anti-tumor activity of systemic delivered formulated miR-29b mimics. Xenografted mice were randomized to receive synthetic miR-29b or miR-NC (1mg/kg per mouse), each formulated with NLE particles, *via* tail vein. Following 3 injections (3 days apart), a significant anti-tumor effect of NLE-formulated miR-29b was detected (Fig. 5C). We observed significant tumor growth inhibition (p<0.05) in mice treated with miR-29b mimics, together with 2-fold increase of miR-29b levels (Fig. 5D) and down-regulation of both DNMT3A and DNMT3B mRNA levels (Fig. 5E) as assessed by qRT-PCR analysis of the excised tumors. Notably, miRNAs administration did not produce any significant behavioral changes or weight loss in treated animals. No pathologic features were detected by histological analysis of tissues, including liver, lung, kidney and bone marrow of treated mice, clearly indicating absence of acute toxicity (not shown).

**Figure 6:**
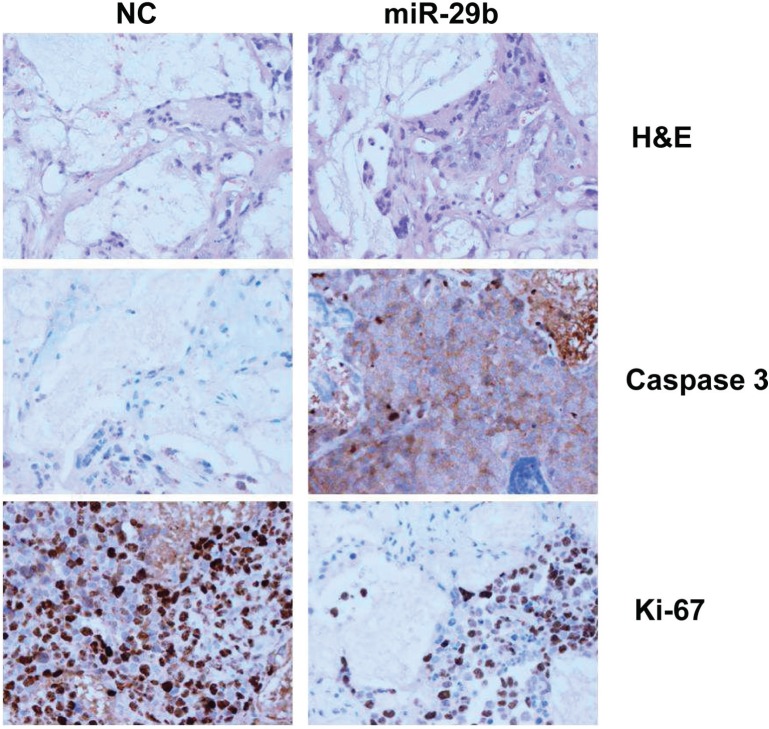
In vivo analysis of miR-29b-effects in the SCID-synth-hu model INA-6 cells were injected in synthetic recipients following engraftment of BMSCs. NLE-formulated miR-29b or NC were injected in groups of three animals, after detection of sIL6R in the mouse sera. Representative Histology (H&E), Caspase-3 and Ki-67 immunohistochemical staining of retrieved 3D biopolymeric scaffolds from treated animals is shown. Original magnification, x40.

Finally, we evaluated the effect of miR-29b mimics in SCID-*synth-hu* mice [29]. In this model, MM cells adhere to BMSCs within a 3D biopolymeric scaffold into immunocompromised mice, recapitulating MM growth within an adult BM MM-derived *milieu*. We used the bone marrow-dependent INA-6 cells to evaluate if miR-29b overcome the growth-supportive effect induced by the huBMM. Histological and immunohistochemical analysis of retrieved 3D biopolymeric scaffolds after treatment with synthetic miR-29b showed indeed increased expression of cleaved caspase-3 and reduced Ki-67 (Fig. 6).

All together, these findings indicate that miR-29b exerts anti-tumor activity *in vivo*, providing a strong rationale for clinical development of synthetic miR-29b mimics in MM.

## DISCUSSION

A number of complex and interdependent epigenetic modifications are involved in cancer development and progression [34]. Alteration of DNA methylation has been reported in hematologic malignancies like acute myeloid leukemia [35] as well as in solid tumors [36, 37], which often display increased levels of DNMTs compared to their normal counterpart, suggesting that DNMTs overexpression contributes to tumorigenesis [35]. In the present work, we show that *de novo* DNMTs mRNA levels are increased in MM and PCL primary samples, indicating a potential role of altered DNA methylation in myelomagenesis. Most importantly, we provide the first evidence that the huBMM increases *de novo* DNMTs expression and therefore could interfere with epigenetic control of MM cell growth and survival.

MiRNAs recently emerged as key players in MM pathophysiology [26]. To investigate the relationship between the miRNA network and *de novo* DNMTs in MM, we studied the regulatory role of miR-29b on DNMTs expression and global DNA methylation in MM cells. Indeed, bioinformatic analysis revealed that DNMT3A and DNMT3B are miR-29b targets, according with previous studies which reported the ability of members of the miR-29-family to target DNMTs in solid tumors and AML [24, 38]. Interestingly, the integrated analysis of miRNA/mRNA profiling revealed an inverse correlation between miR-29b and DNMT3B in a panel of 17 MM cell lines, underscoring a key role of miR-29b on DNMT3B regulation.

We here demonstrate that synthetic miR-29b mimics specifically bind the 3'UTR of DNMT3A and DNMT3B, resulting in downregulation of both DNMTs at mRNA and protein level; conversely, miR-29b inhibition by antagomiRs led to increased DNMTs expression levels. Notably, miR-29b mimics resulted in epigenetic changes, as demonstrated by an approximately 2-fold decrease in global DNA methylation in MM cells. At our knowlewdge, this is the first evidence that a microRNA is able to affect the methylation profile in MM cells. Our results are in line with previous reports showing that enforced expression of miR-29b reduces global DNA methylation in AML and NSCLC cells, restoring the expression of methylation-silenced tumor suppressor genes (TSGs). A variety of TSGs, i.e. VHL, XAF1, IRF8, SOCS1, SOCS3 and CDKN2A, have been found to be promoter-hypermethylated in MM cells. TSGs hypermethylation confers an adverse outcome in MM patients [39-43]. A follow-up study will shade light on TSGs under miR-29b regulation by DNA-methylation control.

In the present report, we also provide insights into biological effects triggered by synthetic miR-29b mimics *in vitro* and *in vivo*. Tumor suppressive role of miR-29b has been previously reported in solid tumors [24, 44] and haematologic malignancies [45], although it remains controversial in CLL [46]. In our experimental model, *in vitro* transfection of synthetic miR-29b mimics in MM cells promoted apoptosis and cell cycle perturbations, and similar effects were observed when DNMT3A and DNMT3B were silenced by shRNAs, just confirming the thought that these enzymes are valuable targets for anti-tumor treatment. Moreover, synthetic miR-29b mimics potentiated the growth-inhibitory effects of the DNMT-inhibitor 5-azacitidine [47], suggesting new strategies based upon DNA-demethylating agents/miRNAs combination in the treatment of MM. The *in vivo* anti-tumor potential of miR-29b was demonstrated in different clinically relevant murine models of human MM. Our experimental platform was based on xenografts of MM cells that were exposed to synthetic miR-29b mimics delivered *via* NLE, a novel lipid-based delivery system. In order to additionally support the clinical translation of our experimental approach, we also studied the *in vivo* activity of miR-29b mimics using the novel SCID-*synth-hu* model of human MM [29]. In this biosynthetic and orthotopic model, tumor cells grow within a bone-like 3D biopolymeric scaffold previously engrafted with human BMSCs, recapitulating a human BM *milieu* in SCID mice. In this model, delivery of systemic miR-29b mimics induced significant anti-tumor effects, as demonstrated by immunohistochemical analysis of retrieved scaffolds, demonstrating the ability of miR-29b to overcome the protective role of BMSCs.

In conclusion, we provide evidence that miR-29b controls the methylation profile of MM cells suggesting that miR-29b down-regulation may play a relevant role in MM pathophysiology by reducing TSGs expression. Moreover, miR-29b mimics appear a promising agent in the treatment of MM, alone or in combination with demethylating drugs.

## MATERIALS AND METHODS

### MM Patient samples and cell lines

Bone marrow mononuclear cells (BMMNCs) and primary MM cells from 55 newly diagnosed untreated MM and 8 plasma cell leukemia (PCL) patients following informed consent and University Magna Graecia IRB approval, were isolated by Ficoll-Hypaque density gradient sedimentation and obtained during standard diagnostic procedures after informed consent in accordance with Institutional guidelines. MM patient cells were separated from BM samples by antibody-mediated positive selection using anti-CD138 magnetic activated cell separation microbeads (Miltenyi Biotech, Gladbach, Germany). Plasma cells (PCs) from tonsils of five healthy donors were also purified and included in the study. The purity of the positively selected PCs was assessed by means of morphology and flow cytometry and was > 90% in all cases. The 55 MM employed for microarray analysis were selected on the basis of the representativeness of their molecular characteristics in order to minimize patient selection biases. MM cell lines (SKMM1, NCI-H929, INA-6, CMA-03, CMA-0306, DELTA47, KMS11, KMS12BM, KMS18, KMS20, KMS26, KMS34, LP1, MM.1.144, MM1S, OPM2, U266 and UTMC2) were cultured in RPMI 1640 (Gibco, Life Technologies, Carlsbad, CA) supplemented with 10% fetal bovine serum (Lonza Group Ltd., Switzerland). The IL-6 dependent MM cell Line INA-6 (kindly provided from Dr Renate Burger, University of Erlangen-Nuernberg, Erlangen, Germany) was cultured in the presence of rhIL-6 (R&D Systems, Minneapolis, MN) as described [48, 49]. BMSCs were established as described [50]. 5-Azacitidine was purchased from Sigma Aldrich and dissolved in RPMI medium as indicated by the manufacturer.

### Gene Expression Profiling

Gene expression profiles were analyzed on the GeneChip^®^ Human Gene 1.0 ST chip platform (Affymetrix Inc., Santa Clara, CA). The raw intensity expression values were processed by Robust Multi-array Average (RMA) complete procedure (Irizarry Biostatistics 2003) with the re-mapped Chip Definition Files (CDF) from BrainArray libraries version 15.0.0 available at http://brainarray.mbni.med.umich.edu/Brainarray/Database/Custom CDF/ 15.0.0/entrezg.asp.

### miRNA profiling

MiRNA expression profiling was carried out using Total RNA and Affymetrix GeneChip miRNA 1.0 according to manufacturer's recommended protocol. Expression values were extracted with Affymetrix miRNA QC tool (RMA normalized and log2-transformed).

### In vitro transfection of MM cells with synthetic miR-29b

Synthetic pre-miRNAs were purchased from Ambion (Applied Biosystems). 1×10^6^ cells were electroporated with scrambled (miR-NC) or synthetic pre-miR-29b (miR-29b) at a final concentration of 100nM, using Neon® Transfection System (Invitrogen), with 1050 V, 30 ms, 1 pulse. Cell transfection efficiency was evaluated by flow cytometric analysis of FAM^TM^-dye-labeled synthetic miRNA inhibitor (Invitrogen) transfection.

### Virus Generation and Infection of MM cells

The human DNMT3A and DNMT3B mission shRNA set (Sigma) and the mission nontarget control transduction virus (SHC002V; Sigma) were used to generate lentiviral particles in HEK 293T packaging cells as previously described [51]; two rounds of transduction of MM cells in the presence of 8 μg/ml of polybrene (Sigma) were performed. Two days after transduction, transduced cells were selected with 1 μg/ml Puromycin (Sigma). Transduced cells underwent two rounds of infection (8 hours each round) and were selected in medium containing 1 μg/ml puromycin. To obtain MM cells stably expressing antagomiR-29b, we used the lentiviral vector miRZip29b anti-miR-29b construct (System Biosciences); lentiviral particles production and transduction were performed according to the above indicated protocols. Selection was performed in medium containing 1 μg/ml puromycin.

### Quantitative real-time amplification of miRNAs and mRNAs

Total RNA from MM cells was prepared with the TRI*z*ol® Reagent (Invitrogen) according to manufacturer´s instructions. The single-tube TaqMan miRNA assays (Applied Biosystems, Assay id 000413) was used to detect and quantify mature miR-29b according to the manufacturer's instructions, by the use of iQ5 multicolor detection system (Bio-Rad). miR-29b expression was normalized on RNU44 (Applied Biosystems, Assay Id 001094). For mRNA dosage studies, oligo-dT-primed cDNA was obtained using the High Capacity cDNA Reverse Transcription Kit (Applied Biosystems) and then used as template to quantify DNMT3A (Hs01027166_m1) and DNMT3B (Hs00171876_m1) levels by TaqMan assay (Applied Biosystems); normalization was performed with GAPDH (Hs03929097_g1). Comparative real-time polymerase chain-reaction (RT-PCR) was performed in triplicate, including no-template controls. Relative expression was calculated using the comparative cross threshold (Ct) method [52].

### Luciferase reporter experiments

The 3' UTRs of DNMT3a and DNMT3B were cloned in pEZX-MT01 vector and purchased from Genecopoeia. MM cells were electroporated as above described using 10μg of the firefly luciferase report; for each plate, 100 nM of the synthetic miR-29b or miR-NC were used. Firefly and Renilla luciferase activities were measured consecutively using the dual-luciferase assay kit (Promega) 24 hours after transfection.

### DNA extraction and measurement of global DNA methylation levels

DNA was extracted by a commercial kit (Invitrogen). The global DNA methylation levels of MM cell lines transfected with syntehtic miR-29b mimics or scrambled oligonucleotides (NC) were estimated according to our previous report [33]. The data were expressed as Global DNA Methylation Index (GDMi) by dividing the mean net luminescence values for the HpaII enzyme to the mean net luminescence values for the MspI enzyme. Thus, the GDMi values inversely correlate to the global DNA methylation levels.

### Cell proliferation and cell cycle analysis

For cell proliferation analysis, MM cells were plated in 6 well plates, electroporated with miR-29b or miR-NC and then harvested, plated at 1,5-2,0×10^5^/mL, and counted at 24-hour intervals using a Trypan Blue-exclusion assay. Each sample was run at least in triplicate. Cell cycle analysis was performed as previously reported [53].

### Western blotting

SDS-PAGE and Western Blotting (WB) were performed according to standard protocols[35]. Briefly cells were lysed in lysis buffer containing 15mM Tris/HCl pH 7.5, 120mM NaCl, 25mM KCl, 1mM EDTA, 0.5% Triton 100, Halt Protease Inhibitor Single-Use cocktail (100X, Thermo Scientific). Whole cells lysates (50μg per lane) were separated using 4-12% Novex Bis-Tris SDS-acrylamide gels (Invitrogen), electro-transferred on Nitrocellulose membranes (Bio-Rad), and immunoblotted with the rabbit anti-DNMT3A, anti-DNMT3B or anti-GAPDH (Santa Cruz Biotechnology) antbodies.

### Animals and in vivo models of human MM

Male CB-17 severe combined immunodeficient (SCID) mice (6- to 8-weeks old; Harlan Laboratories, Inc., Indianapolis) were housed and monitored in our Animal Research Facility. All experimental procedures and protocols had been approved by our Institutional Ethical Committee and conducted according to protocols approved by the National Directorate of Veterinary Services (Italy). Procedures were performed as previously described [53]. Briefly, mice were sacrified when their tumors reached 2 cm in diameter or in the event of paralysis or major compromise in their quality of life, to prevent unnecessary suffering, as previously described [54]. For our study, we used 2 different models of human MM, including i) SCID mice bearing subcutaneous (sc) MM xenografts [55]; and ii) SCID mice implanted with a 3D polymeric scaffold previously reconstituted with human bone marrow stromal cells and then injected with INA6 human MM cells (SCID-synth-hu)[30].

#### i) Xenograft of MM cell lines in SCID mice

For this model, mice were sc inoculated in the interscapular area with 5 × 10^6^ MM cells cells in 100 μL RPMI-1640 medium. Treatment was initiated after the detection of palpable tumors, approximately 3 weeks following injection of MM cells. Tumor sizes were measured weekly in 2 dimensions using a caliper, and volume was calculated using the formula: V = 0.5 × a × b^2^, where a and b are the long and short diameter of the tumor, respectively, until the tumor was palpable. Mice were randomized in 3 groups and treated with synthetic miR-29b mimics or miR-NC or vehicle alone or PBS. Each dose contained 20 μg synthetic oligo which equals 1mg/kg per mouse with an average weight of 20g. Administration of miRNA-mimics was performed by the use of the novel formulation of neutral lipid emulsion (NLE) (MaxSuppressor *in vivo* RNA Lancer II, BIOO Scientific, Austin, TX) according to the manifacturer's instructions. Treatments were first performed intratumorally (i.t.) every three days for a total of four injections. In a subsequent series of experiments, treatments were performed systemically via tail vein by using the same formulation and dosage/schedule of the intratumoral injections. Tumors were then collected and placed in either 10% formalin for histology or in RNA*later*® for RNA isolation.

#### ii) SCID-synth-hu model

The SCID-*synth-hu* model was reproduced by implantation into a SCID mouse of a three-dimensional (3D) bone-like poly-Σ-caprolactone polymeric scaffold (PCLS)[29]. PCLS cylinders (7mm in length and 3mm in diameter at scanning electron microscope SEM), demonstrated interconnected large (100–300 mm) and small pores (1–10 mm) resembling the micro-architecture of a normal human adult bone, as described. Dynamic seeding of BMSCs into PCLSs was performed as previously reported. Briefly, using a suspension of 8×10^5^ cells in 500 μl of growth medium, a 22-gauge needle on a 2.5 ml syringe was threaded into two ending faces of the cylindrical scaffold. A medium flow rate of 500 μl/min and three drawing cycles were carried out on both scaffold ends. Before implantation, PCLSs were incubated in complete medium at 37°C in 5% CO2 for 24 h to allow cell adhesion on 3D surfaces. Then, the PCLS was surgically implanted sc into a SCID mouse flank. Chloralium hydrate anesthesia (400 mg/kg, 0,15 ml) was used during all surgical procedures. Three weeks thereafter, 8×10^5^ bone marrow-dependent INA-6 cells were injected *in vivo* into previously implanted PCLSs coated *ex vivo* with human derived BMSCs. Approximately one month later, when sIL6R became detectable in mice sera, NLE-miR-29b or -NC were injected directly into the scaffold (total of 7 injections, 2 days apart). *In vivo* effects induced by miR-29b were evaluated by immunohistochemistry on retrieved scaffolds at the end of treatments.

### Histology and immunohistochemistry

Retrieved tumors from animals were immediately fixed by immersion in 4% buffered formaldehyde for 24 h at 4 °C, washed, dehydrated and embedded in paraffin. For the light microscopy analysis, sections were cut (4 μm), mounted on poly-lysine slides, and stained with H&E. For immunohistochemistry staining, 2μm thick sections were in a Bond Max Automated Immunohistochemistry according to the following protocol. Tissues were deparaffinized and pre-treated with the Epitope Retrieval Solution 2 (EDTA-buffer pH8.8) at 98°C for 20 min. After washing steps, peroxidase blocking was carried out for 10 min using the Bond Polymer Refine. Tissues were again washed and then incubated with the primary antibody directed against ki67 (Dako, clone: MIB-1; 1:150), capase-3 (NOVOCASTRA, clone: JHM62; 1:500). Subsequently, tissues were incubated with polymer for 10 min and developed with DAB-Chromogen for 10 min. Slides were counterstained with hematoxylin.

### Statistical analysis

Student's *t* test, two-tailed and Log rank test were used to calculate all reported *P* values using GraphPad software (www.graphpad.com). Graphs were obtained using SigmaPlot version 11.0.

## Supplementary Figures


