# Supplementary material for: DNA-demethylating and anti-tumor activity of synthetic miR-29b mimics in multiple myeloma
Source: Oncotarget. 2012 Oct 21;3(10):1246–58. doi: 10.18632/oncotarget.675 (PMC3717964; doi:10.18632/oncotarget.675)
Supplement: Supplementary file 1 [file oncotarget-03-1246-s001.pdf]

DNA-demethylating and anti-tumor activity of synthetic miR-29b mimics in multiple myeloma - Amodio et al

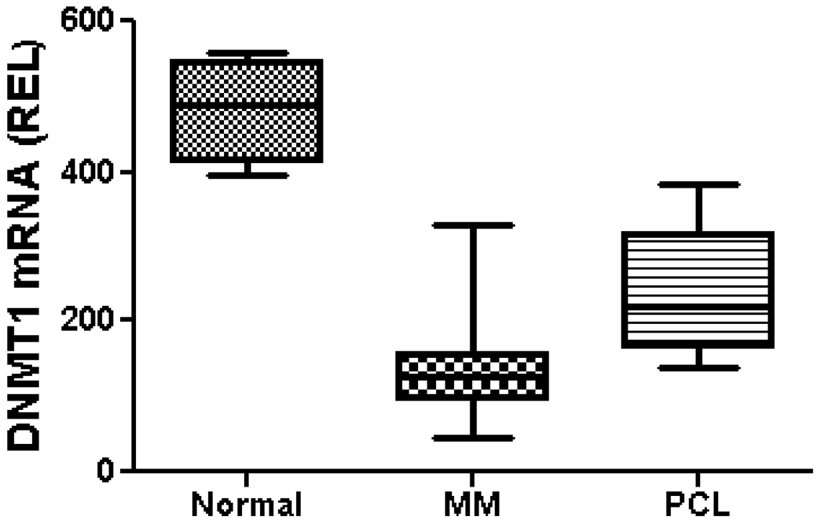

Figure S1: Expression of DNMT1 in MM and PCL patients. Differential expression of DNMT3A (A) and DNMT3B (B) in controls, MM and PCL patients. DNMT3A and DNMT3B mRNA levels were obtained by cDNA microarray and reported as raw expression values. The statistical significance of differences among the groups was assessed using Kruskal-Wallis test (P<0,001).

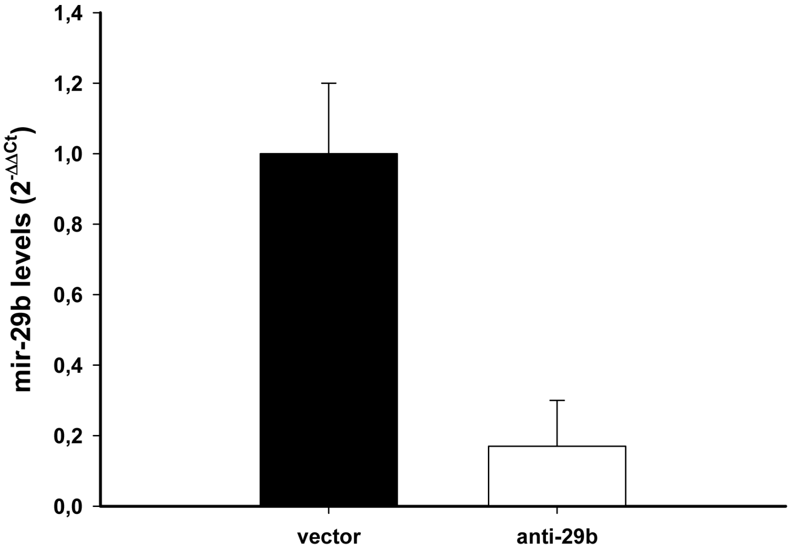

Figure S2: MiR-29b levels in SKMM1 cells transduced with antagomiR-29b. Quantitative RT-PCR of miR-29b levels in SKMM1cells transduced with lentivirus carrying antagomiR-29b (antimiR-29b) or the empty vector. Raw Ct values were normalized to RNU44 housekeeping snoRNA and expressed as ΔΔCt values. MiR-29b levels in cells transduced with the empty vector were set as internal reference.

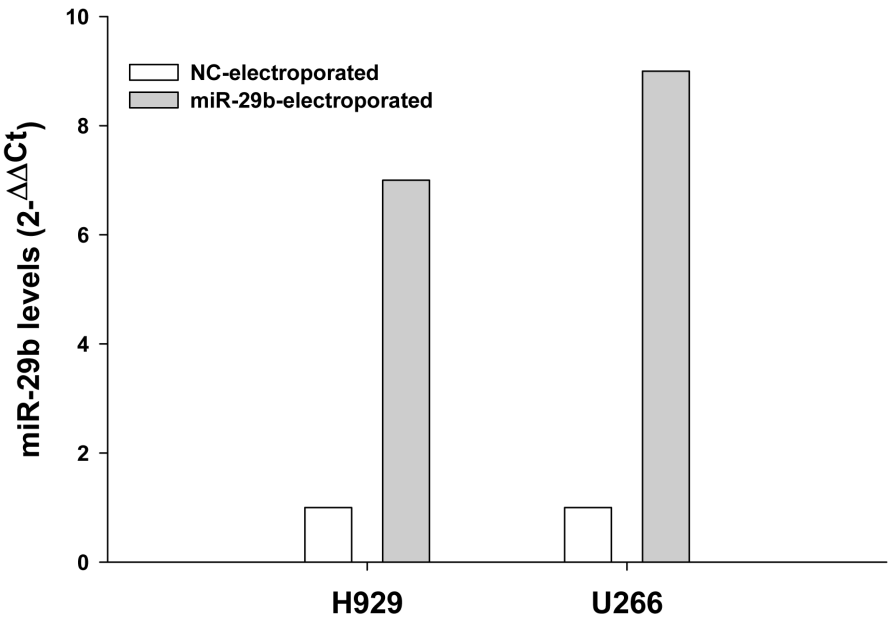

Figure S3: MiR-29b levels in electroporated U266 and NCI-H929 cells. Quantitative RT-PCR of miR-29b levels in U266 and NCI-H929 cells transfected with synthetic miR-29b mimics or scrambled oligonucleotides (NC). Raw Ct values were normalized to RNU44 housekeeping snoRNA and expressed as ΔΔCt values. MiR-29b levels in cells transfected with NC were set as internal reference for each cell line. Data are the average of two independent transfection experiments performed in triplicate.

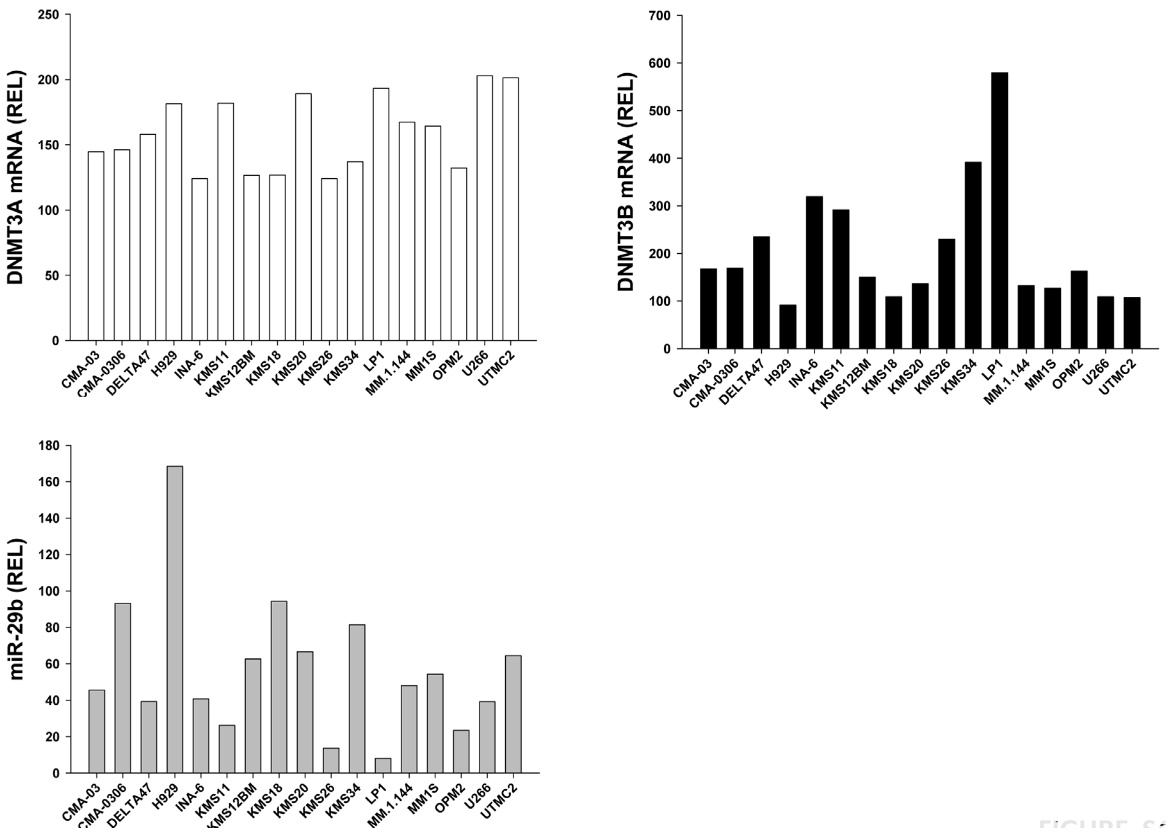

Figure S4: DNMT3A, DNMT3B and miR-29b expression levels in MM cell lines. DNMT3A, DNMT3B and miR-29b were profiled by microarray as described in Materials and Methods. Raw expression levels are reported in graph.

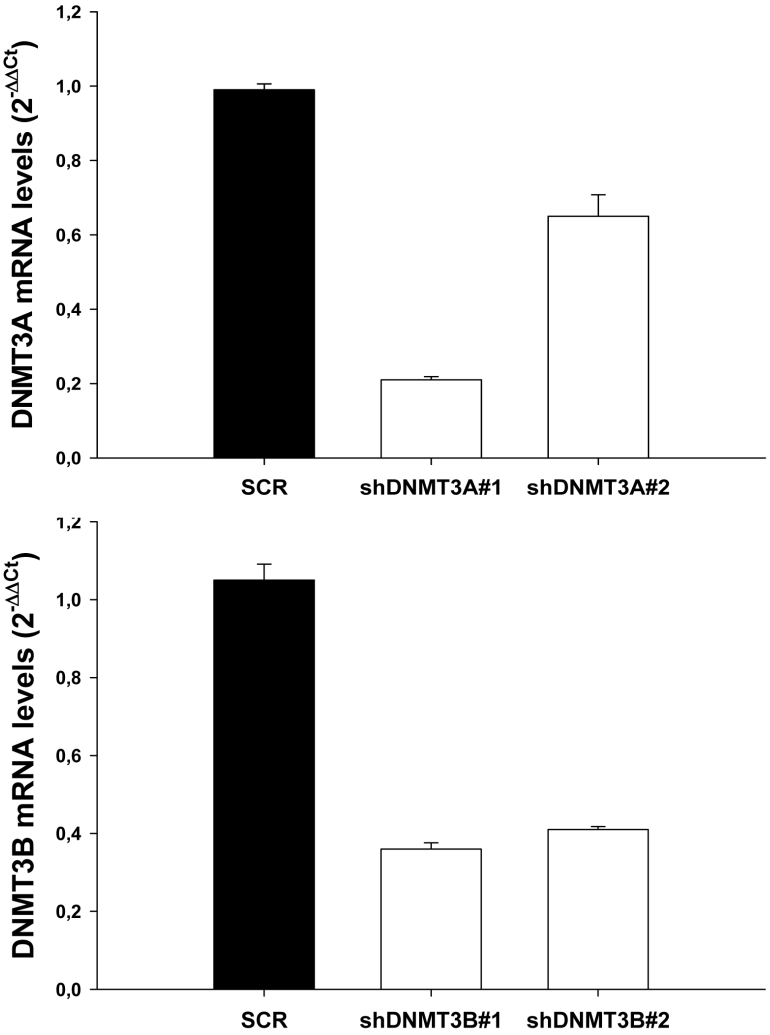

Figure S5: DNMT3A and DNMT3B mRNA levels after transduction of NCI-H929 with shRNAs. NCI-H929 were transduced with two different shRNAs against DNMT3A or DNMT3B. After transduction and puromycin selection, as detailed in Materials and Methods, expression levels of DNMT3A or DNMT3B were assessed by quantitative-RT-PCR. Raw Ct values were normalized to GAPDH and expressed as ΔΔCt values calculated using the comparative cross threshold method. DNMT3A or DNMT3B levels in cells transduced with the scrambled vector were set as internal reference.
